# A Microfluidic Fluorescent Flow Cytometry Capable of Quantifying Cell Sizes and Numbers of Specific Cytosolic Proteins

**DOI:** 10.1038/s41598-018-32333-1

**Published:** 2018-09-21

**Authors:** Xiufeng Li, Beiyuan Fan, Lixing Liu, Deyong Chen, Shanshan Cao, Dong Men, Junbo Wang, Jian Chen

**Affiliations:** 1 State Key Lab of Transducer Technology, Institute of Electronics of Chinese Academy of Sciences, Beijing City, China; 2 University of Chinese Academy of Sciences, Beijing City, China; 3 State Key Lab of Virology, Wuhan Institute of Virology of Chinese Academy of Sciences, Wuhan City, Hubei Province China

## Abstract

This study presents a microfluidics based cytometry capable of characterizing cell sizes and counting numbers of specific cytosolic proteins where cells were first bound by antibodies labelled with fluorescence and then aspirated into a constriction microchannel in which fluorescent levels were measured. These raw fluorescent pulses were further divided into a rising domain, a stable domain and a declining domain. In addition, antibody solutions with labelled fluorescence were aspirated through the constriction microchannel, yielding curves to translate raw fluorescent levels to protein concentrations. By using key parameters of three domains as well as the calibration curves, cell diameters and the absolute number of β-actins at the single-cell level were quantified as 14.2 ± 1.7 μm and 9.62 ± 4.29 × 10^5^ (A549, n_cell_ = 14 242), 13.0 ± 2.0 μm and 6.46 ± 3.34 × 10^5^ (Hep G2, n_cell_ = 35 932), 13.8 ± 1.9 μm and 1.58 ± 0.90 × 10^6^ (MCF 10 A, n_cell_ = 16 650), and 12.7 ± 1.5 μm and 1.09 ± 0.49 × 10^6^ (HeLa, n_cell_ = 26 246). This platform could be further adopted to measure numbers of various cytosolic proteins, providing key insights in proteomics at the single-cell level.

## Introduction

Quantitative analysis of single-cell protein expressions can provide information in understanding heterogeneities of cells within the fields of immunology and oncology^1–3^. Nowadays, flow cytometers are the golden instruments for quantifying protein numbers at the single-cell level in which cells bound with antibodies labelled with fluorescent or isotope probes travel rapidly through a detection region with corresponding fluorescent levels or isotope numbers measured^4–6^. Based on calibrating microbeads, flow cytometers enable absolute counting of membrane proteins of single cells^7–10^, pushing forward the developments of various diseases involving white and red cells^5^. However, when conventional flow cytometers are leveraged to estimate cytosolic proteins for deep phenotyping^11,12^ and signaling state characterization^13–16^, they are incapable of collecting numbers of specific cytosolic proteins since the corresponding calibration microbeads are missing, severely compromising developments in these fields^1–3^.

Microfluidics is a technology of processing fluids based on microchannels with critical geometries of tens to hundreds of μm^17,18^. Due to the dimensional comparisons between microfluidics and biological cells, microfluidics has functioned as an enabling platform for single-cell protein analysis^19,20^. Currently, microfluidic platforms for single-cell protein analysis are divided into miniaturized flow cytometers^21–23^ and microfabricated arrays (e.g., microengraving^24–28^, barcoding microchips^29–32^, western blot of single cells^33^ and microwells for single-cell isolation and characterization^34–37^).

Among these developed microfluidic platforms, microengraving and barcoding microchips can realize absolute measurements of specific cytosolic proteins, by confining single cells in microfabricated domains with targeted proteins captured by antibodies previously coated within the detection areas^19,20^. However, compared to flow cytometers, these microfluidic approaches have lower throughputs since they are not capable of processing cells continuously. As to the miniaturized flow cytometry, due to the lack of calibration beads, counting of specific cytosolic proteins was not reported by the majority of micro flow cytometry^21–23^. Recently, a modified fluorescent micro flow cytometry was reported, enabling the translation of raw fluorescent signals into specific protein concentrations, which, however, cannot be further translated to absolute numbers due to the lack of the critical information of cell sizes^38^.

With the purpose of dealing with this problem, this manuscript reports a constriction microchannel based flow cytometer capable of simultaneously characterizing cellular sizes and specific cytosolic proteins. In the modified flow cytometry, cells bound with antibodies labelled with fluorescent probes are deformed through the constriction microchannel with cross-sectional areas smaller than cells where profiles of fluorescence are collected as a function of time, which are further processed to obtain cellular sizes and raw fluorescent intensities. In addition, fluorescent antibodies are aspirated through the constriction microchannel to produce calibration curves. Based on cell sizes, preliminary fluorescent intensities as well as the calibrating curve, counting of specific cytosolic proteins at the single-cell level can be obtained. Compared to well-established flow cytometers, this platform can provide a calibrating strategy of translating preliminary signals into protein numbers. In comparison to other microfluidic systems (e.g., barcoding microchips and microengraving), this study can enable the counting of single-cell cytosolic proteins in a high-throughput manner.

## Materials and Methodologies

### Materials

If not specifically mentioned, reagents for cell cultures were bought from Life Technologies (USA). Materials used for cellular processing (e.g., protein fixation, membrane penetration, anti-fouling block and intracellular staining) mainly include triton X-100 and bovine serum albumin (BSA) from Sigma-Aldrich (USA) as well as anti β-actin antibody from ABCAM (UK). Materials for microfabrications include photoresist of SU-8 from MicroChem (USA) and elastomer of 184 silicone from Dow Corning (USA).

### Working Principle

**Figure 1:**
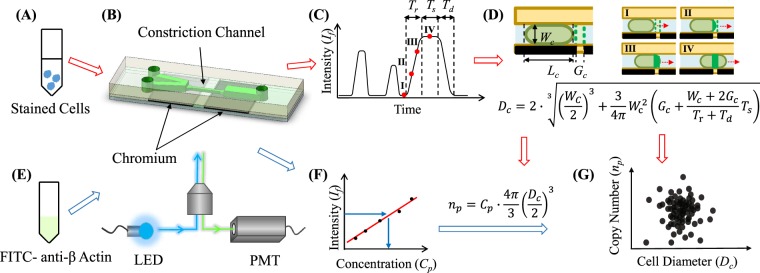
The schematic of the constriction microchannel based microfluidic flow cytometry enabling the simultaneous characterization of cellular sizes and absolute numbers of specific cytosolic proteins. Cells stained with fluorescence labelled antibodies (**A**) were flushed into the constriction microchannel (**B**) with excited fluorescence detected by a photomultiplier tube (**C**). (**D**) The fluorescent profile of a representative cell can be divided into a rising domain of *T*_*r*_, a stable domain of *T*_*s*_ with a fluorescent level of *I*_*f*_ and a declining domain of *T*_*d*_. The corresponding stages of the travelling cell are also included in (**D**) in which the deformed cell shows an elongation length of *L*_*c*_, and the middle portion of the deformed cell fully fills the constriction microchannel and both the front and rear surfaces are modelled as semi-spheres with diameters the same as the width of constriction microchannel as *W*_*c*_. Due to the constriction microchannel design, by flushing solutions with a group of concentrations of fluorescence labelled antibodies (**E**) into the constriction microchannel with fluorescent intensities recorded, the calibration curve (**F**) can be obtained. Based on raw parameters of *T*_*r*_, *T*_*s*_, *I*_*f*_, and *T*_*d*_, as well as the calibration curve, cell diameters (*D*_*c*_) and concentrations of the targeted proteins (*C*_*p*_) per cells were quantified, leading to the absolute numbers of the specific protein (*n*_*p*_) at the single-cell level (**G**).

The developed microfluidic flow cytometer is mainly composed of a constriction microchannel plus a microfabricated chrome window as the detection domain of fluorescence, a LED (light emitting diode) based light source and a PMT (photomultiplier tube) based fluorescent detector (please refer to Fig. 1).

In experiments, individual cells bound with antibodies labelled with fluorescent probes (refer to Fig. 1(A)) are deformed into the constriction microchannel (refer to Fig. 1(B)) and the excited fluorescent signals are captured by PMT (refer to Fig. 1(C)). Because the constriction microchannel is smaller than cells, cells are forced to deform through the constriction microchannel with an elongation length *L*_*c*_, where the middle portion of the deformed cell fully fills the constriction microchannel^38^ while both the front and rear surfaces are treated as semi-spheres with diameters the same as the width of constriction microchannel (*W*_*c*_) (refer to Fig. 1(D)).

The fluorescent profile of a representative cell as a function of time can be divided into three domains (refer to Fig. 1(D)), which includes a rising domain with a time duration of *T*_*r*_, a stable domain with a fluorescent level of *I*_*f*_ and a time duration of *T*_*s*_ and a declining domain with a time duration of *T*_*d*_. In the rising domain, there is a gradual increase of fluorescent intensities indicating that the deformed cell with fluorescence labelled antibodies gradually fills the fluorescent detection region defined by the patterned chrome layer where the cellular traveling distance for this domain is mainly the chrome gap (${G}_{c}+{W}_{c}/2$). As the deformed cell further moves in the constriction microchannel, there is a duration with a stable fluorescent intensity of *I*_*f*_, which indicates the full occupation of the constriction microchannel. The time duration *T*_*s*_ of this section corresponds to the travelling distance of cell elongation minus the width of the constriction microchannel (${L}_{c}-{W}_{c}-{G}_{c}$). As for the declining domain with a duration of *T*_*d*_, it corresponds to the gradual decrease in fluorescent intensities for the leaving of the deformed cell with the same travelling distance as ${G}_{c}+{W}_{c}/2$. Based on this analysis, the cell elongation length of *L*_*c*_ can be obtained by interpreting the fluorescent profile as a function of time, which is further translated to the diameters of cells *D*_*c*_ (refer to Fig. 1(D)).

Due to this unique design of cellular full occupations of the constriction microchannel, varied concentrations of antibodies labelled with fluorescent probes are flushed into the constriction microchannel where fluorescent signals are measured to form calibrating curves, illustrating the relation between intensities of fluorescence and concentrations of specific proteins (refer to Fig. 1(F)). Based on raw parameters of *T*_*r*_, *T*_*s*_, *I*_*f*_, and *T*_*d*_, as well as the calibration curve, cell diameters (*D*_*c*_) and concentrations of the targeted protein (*C*_*p*_) per cells were quantified, resulting to the number counting of the targeted protein at the single-cell level (refer to Fig. 1(G)).

### Culture, Processing and Characterization of Biological Cells

All cell types were bought from the China Infrastructure of Cell Line Resources (China) and the cell culture was conducted with an incubator of Thermo Scientific (USA) under the conditions of 37 °C and 5% CO_2_. The adenocarcinomic human alveolar basal epithelial cell type of A549 was seeded in a RPMI-1640 medium plus 10% w/w FBS and 1% w/w antibiotics. The human hepatocellular tumorigenic cell type of Hep G2 was seeded in a DMEM medium plus 10% w/w FBS and 1% w/w antibiotics. The non-tumorigenic human breast epithelial cell type of MCF 10 A was seeded in a DMEM/F-12 medium plus NEAA (1% w/w), HS (5% w/w), insulin (10 μg/ml), EGF (20 ng/ml), cholera toxin (100 ng/ml), hydrocortisone (0.5 μg/ml), and antibiotics (1% w/w). The human cervical tumour cell type of HeLa was seeded in a DMEM medium plus 10% w/w FBS and 1% w/w antibiotics. Prior to experiments, adherent cells in the logarithmic phase (e.g, 24 hours after seeding of A549 and HeLa cells and 48 hours after seeding of Hep G2 and MCF 10 A cells) were trypsinized, centrifuged and resuspended in phosphate buffer saline supplemented with 0.5% bovine serum albumin at a concentration of ~10 million cells per mL.

Intracellular staining of β-actins was realized by using conventional protocols widely used in the experiments of flow cytometers, including major steps of protein fixation and immoblization, membrane penetration and permeabilization, anti-fouling blocking and staining of antibodies. Within this experiment, in the first step of fixation, the cells in suspensions were incubated with 2% formaldehyde (at 4 °C for a duration of 15 min). Then, the cells were incubated with triton X-100 for 15 min at 4 °C with membranes punched with holes. As to percentages of triton X-100, a variety of concentrations were used for comparisons, which were 0.05%, 0.1% and 0.3% for A549 cells; 0.01%, 0.03%, 0.05% and 0.07% for Hep G2 cells; 0.05%, 0.10%, 0.15% and 0.20% for MCF 10 A cells; and 0.05%, 0.10%, 0.15% and 0.20% for HeLa cells.

Then anti-fouling blocking was done using 5% BSA at the room temperature for a duration of 30 min. Furthermore, β-actin antibodies with fluorescent probes at a dilution of 1:100 were applied for cell incubations at a group of time durations (e.g., 1 hour, 2 hours, 4 hours, or 8 hours) under the condition of 37 °C.

To evaluate the results of cellular staining, both bright-field and fluorescent images of stained cells were collected based on a fluorescent microscopy and a CCD (IX 83 and DP73, Olympus, Japan). Stained cells were added on a glass slide and confined to the height of 100 μm with the help of a second cover slide. After placing the slides on the microscope, bright-field and fluorescent pictures were recorded at an exposure time of 40 ms to alleviate the concerns of photobleaching.

Based on bright-field images, the status of cellular aggregations due to excessive membrane penetrations was evaluated to optimize the percentages of triton x-100 used in the step of membrane permeabilization. ImageJ (National Institute of Health, USA) was used to process fluorescent images and extract cellular pixel intensities to optimize the time durations in the step of antibody staining.

### Design and MicroFabrication of Constriction Microchannels

Within this platform, constriction microchannels (8 μm in width and 8 μm in height) with 2.5 μm gaps made of chrome were fabricated to count specific type of cytosolic proteins at the single-cell level. The geometries of the constriction microchannel ensure that tumour cells (10–18 μm in diameter) could effectively deform and completely fill the constriction microchannel. Furthermore, at the inlet of the constriction microchannel, a gradual decrease in the channel width was used in this study with the corresponding values changed from 25 μm to 8 μm, decreasing the possibilities of channel blockage by incoming large cells.

Furthermore, the choice of the width of the chrome gap can also affect the diameters of cells that can be measured. In order to quantify cellular diameters, the process of cellular travelling through the chrome gap has to be decoupled into the rising domain, the stable domain and the declining domain. If the gap of the chrome window is too big, small cells cannot fully occupy the detection room, resulting in misleading data. In order to address this issue, 2.5 μm, the minimal feasible gap of chrome based on microfabrication was chosen in this study.

The proposed device was fabricated based on conventional microfabrication techniques in which the patterned PDMS layer including constriction microchannels was peeled away from a SU-8 mold based on twice photolithography. Meanwhile, a layer of chrome was first deposited on a quartz slide, which was further patterned to form gaps and then coated with a thin layer of unpatterned PDMS. Then the patterned PDMS layer and the glass layer with patterned chrome gaps were bonded together after plasma treatment (refer to Supp. Figure 1).

Briefly, SU-8 was spun on a glass slide, exposed without development to form the constriction microchannel (refer to Supp. Figure 1(A)). Then, SU-8 was spun again on the previous layer of photoresist, exposed with alignments and developed to form channels for cell transportations (refer to Supp. Figure 1(B,C)). Monomers and crosslinking agents of PDMS (10:1 by weight) were thoroughly mixed with bubbles removed, and casted on SU-8 molds, which were then put in an oven for crosslinking (overnight at 80 °C). After full crosslinking, the PDMS layers were removed from the mold of SU-8 and a metal puncher was used to form channel inlets and outlets (refer to Supp. Figure 1(D,E)).

Fabrications of chrome windows were realized by conventional microfabrication technologies including chrome sputtering on a quartz slide (refer to Supp. Figure 1(F)), micropatterning of photoresist (refer to Supp. Figure 1(G)), etching of chrome (refer to Supp. Figure 1(H,I)) and coating of an ultra-thin PDMS layer without patterning (refer to Supp. Figure 1(J)). More specifically, monomers and crosslinking agents of PDMS (10:1 by weight) were dissolved in hexane (1:16), thoroughly mixed, degassed, and spin coated on the patterned quartz slides to form a thin PDMS layer with a thickness of 0.8–1 μm. Finally, following the treatment of oxygen plasma, the PDMS layer with constriction microchannels and the chrome layer with microfabricated windows were sealed together (refer to Supp. Figure 1(K,L)).

### Platform Operations and Result Analysis

In experiments, the constriction microchannel based microfluidic flow cytometry was rinsed with phosphate buffer saline with 0.5% bovine serum albumin and positioned on a fluorescent microscope (IX 83, Olympus, Japan) for photobleaching of 20 min. In this fluorescent microscope, the LED (M470L3-C1, Thorlabs, USA) with a wavelength of 470 ± 40 nm and an output power of 150 mW was used as the light source. Then stained cells with antibodies labelled with fluorescent probes were aspirated through constriction microchannels using a pressure generator (DPI-610, Druck, UK). Note that different cell types have different distributions of cell diameters and thus aspiration pressures were finely tuned to suck individual cell types through the constriction microchannel, which were 7–10 kPa for A549 cells, 5–7 kPa for Hep G2, 15–20 kPa for MCF 10 A and 15–20 kPa for HeLa cells.

Fluorescence of single cells travelling in the constriction microchannel was measured by the PMT of H10722-01(Hamamatsu, Japan), and then recorded by the DAQ of PCI-6221(NI, USA) at 100 kHz. In the step of calibration, antibody solutions with dilution times of 10, 50, 100, 500, 1000 and 5000 were forced to travel in the constriction microchannels, consistent with experimental conditions of single-cell analysis.

The preliminary data collected by DAQ were further processed based on median filtering (fifty points) to get rid of dark currents generated by PMT. Then, basal signals without cells were represented as average ± standard deviation and electrical pulses with amplitudes higher than average + 3 x standard deviation were regarded as effective events with travelling cells.

These electrical pulses were then divided into three domains based on curve fitting, collecting four key parameters, which are *T*_*r*_, *T*_*s*_, *T*_*d*_ and *I*_f_. Then two intermediate parameters of *D*_*c*_ (cell diameters derived from *T*_*r*_, *T*_*s*_, *T*_*d*_) and *C*_*p*_ (the concentration of β-actins at the single-cell level derived from *I*_f_ and the calibration curve) were quantified and further translated to *n*_*p*_ (the absolute number of single-cell β-actins).

### Statistics

For each experiment, more than three times were conducted and the results were expressed as average ± standard deviation. Comparison of multiple groups were conducted using ANOVA based Turkey Test and P < 0.05 (*) represented significances with statistical meaning.

## Experimental Results with Discussions

As a housekeeping protein, β-actin is as an obligatory part of the cell cytoskeleton, playing key roles in maintenance of cellular shape, migration, division, growth and signalling^39^. Due to their constitutive expressions, β-actins are commonly used as internal controls in western blots based on the assumption that the expression levels of β-actins remain constant from cell to cell, sample to sample, treatment to treatment and patient to patient^40–42^. However, recent studies based on population analysis indicate that the expressions of β-actins can change in response to biochemical stimuli, during growth and differentiation, and in disease states^41,43–49^. Whether there also exists expression differences of β-actins among individual cells remains elusive. Thus, in this manuscript, a flow cytometry leveraging constriction microchannels was developed to quantitatively estimate the numbers of single-cell β-actins.

### Intracellular Staining of β-Actins

With the purpose of counting the numbers of single-cell β-actins, intracellular staining with β-actin antibodies labelled with fluorescent probes was conducted, including major steps such as protein fixation, membrane penetration, anti-fouling block and staining with antibodies. The use of triton X-100 for membrane permeabilization was optimized to ensure intracellular β-actins were fully exposed. Theoretically, a maximal concentration of triton x-100 should be used to fully penetrate cell membranes without compromising cellular integrities. However, it was observed that high concentrations of triton x-100 lead to severe membrane compromises and cellular aggregations in the following step of antibody staining, which can produce uneven staining at the single-cell level. Thus, in this study, the percentages of triton x-100 were finely tuned for individual cell types, producing the optimal values of 0.05% for A549 cells, 0.03% for Hep G2 cells, 0.1% for MCF 10 A cells and 0.1% for HeLa cells.

**Figure 2:**
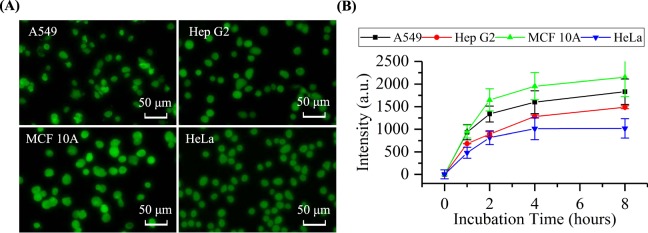
(**A**) Fluorescent pictures of stained A549, Hep G2, MCF 10 A and HeLa cells where the intensities of stained cells were quantified as a function of time (**B**). Four types of cells showed similar trends where the intensities of stained cells were observed to initially increase with the incubation time, and then saturate after four hours of incubation. Further increases in the incubation durations (e.g., eight hours) didn’t lead to further significant increases in the fluorescent intensities, suggesting that after four hours of incubating cells with antibodies, the majority of the exposed cytosolic β-actins were bound with fluorescence labelled antibodies.

Furthermore, in the step of cellular staining with fluorescence labelled antibodies, different incubation times were tested for these four types of cells in order to make sure that the majority of the exposed β-actins were taken by antibodies. As shown in Fig. 2(A), fluorescent images of stained A549, Hep G2, MCF 10 A and HeLa cells were demonstrated where the fluorescent levels of cells after the step of staining were measured as a function of time (refer to Fig. 2(B)).

Four types of cells showed similar trends where the stained fluorescent intensities increased as a function of the incubation time initially, and then saturate after four hours of incubation. Further extensions in the durations of incubation produced no dramatic improvements in the levels of fluorescence, suggesting that the majority of cytosolic β-actins was taken by corresponding antibodies after a four-hour duration of incubations. Note that these fluorescent intensities obtained from microscopic images are “relative” values based on “black balances” of the fluorescent pictures and “zeroing” of the fluorescent intensities of unstained cells of a specific type. Thus, although these fluorescent intensities can demonstrate the trend of antibody staining as a function of time, the obtained fluorescent values of different cell types cannot be effectively compared since they are relative values.

### Collection and Processing of Raw Fluorescent Pulses

**Figure 3:**
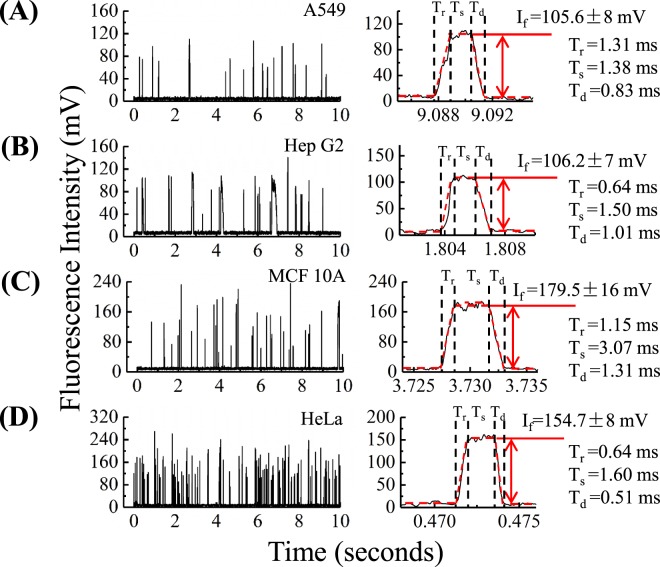
Preliminary fluorescent intensities of A549 (**A**), Hep G2 (**B**), MCF 10 A (**C**) and HeLa (**D**) cells where each pulse represents a passing cell, which was further divided into a rising domain with a time duration of *T*_*r*_, a stable domain with a fluorescent level of *I*_*f*_ and a time duration of *T*_*s*_ and a declining domain with a time duration of *T*_*d*_ based on curve fitting.

Stained cells were then aspirated to deform into the constriction microchannel with raw fluorescent levels recorded in a time sequence. Figure 3(A–D) represent raw characterization results of single A549, Hep G2, MCF 10 A and HeLa cells through the constriction microchannel where individual pulse stands for a travelling cell, which was further divided into the rising domain with a time duration of *T*_*r*_, the stable domain with a fluorescent level of *I*_f_ and a time duration of *T*_*s*_ and the declining domain with a time duration of *T*_*d*_ based on curve fitting.

**Figure 4:**
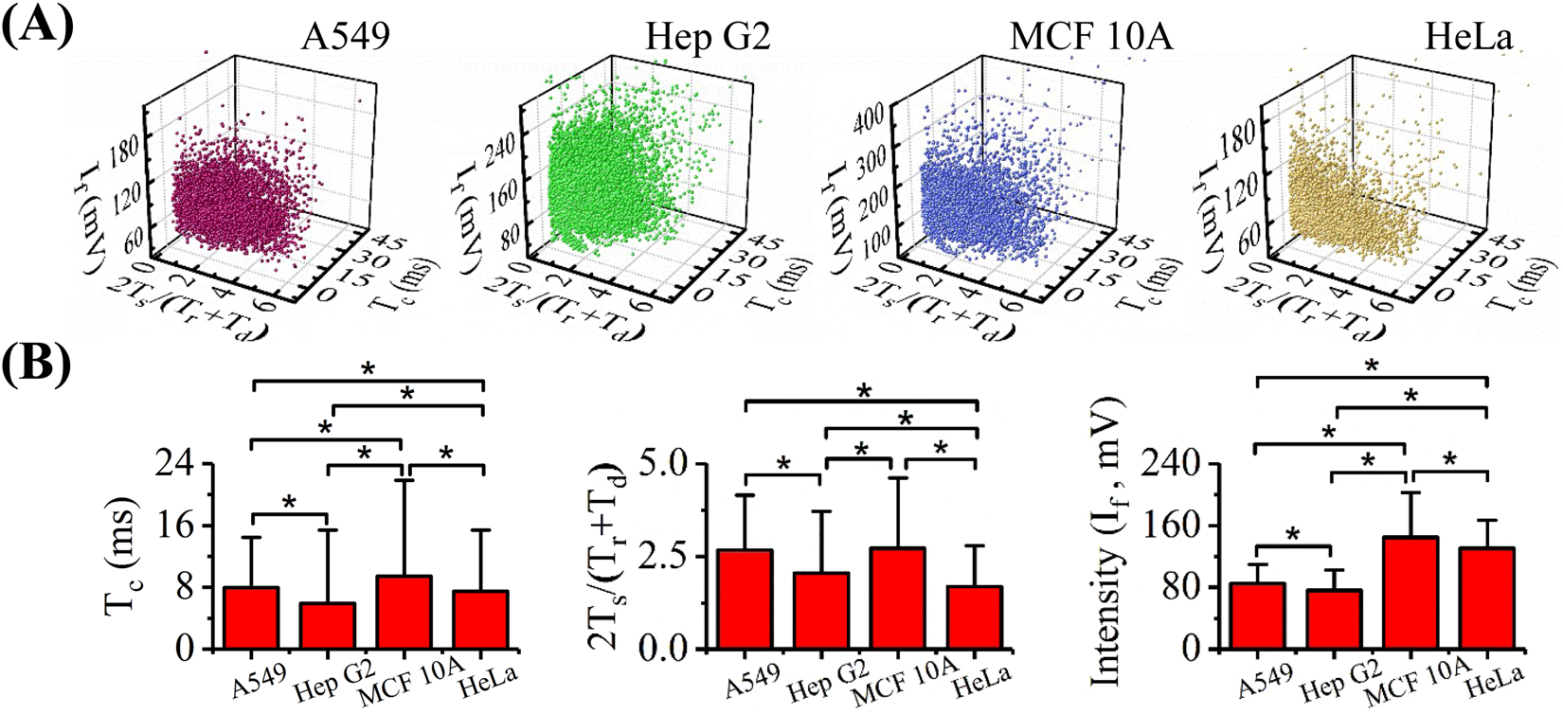
3D scatter plots of *T*_*c*_ (a summary of *T*_*r*_, *T*_*s*_ and *T*_*d*_, indicating travelling durations of individual cells), *I*_*f*_ (an indicator of the concentration of β-actins) and *2T*_*s*_*/(T*_*r*_ + *T*_*d*_*)* (an indicator of cell sizes) for A549 (n_cell_ = 14 242), Hep G2 (n_cell_ = 35 932), MCF 10 A (n_cell_ = 16 650) and HeLa (n_cell_ = 26 246) cells.

Based on the curve fitting of these fluorescent pulses, several key parameters were further processed. First, the total travelling duration of a cell (*T*_*c*_) as a sum of *T*_*r*_, *T*_*s*_, and *T*_*d*_ was calculated, which were 7.8 ± 6.4 ms (A549 cells, n_cell_ = 14 242), 5.7 ± 9.1 ms (Hep G2 cells, n_cell_ = 35 932), 8.7 ± 11.5 ms (MCF 10 A cells, n_cell_ = 16 650) and 7.4 ± 7.7 ms (HeLa cells, n_cell_ = 26 246), respectively. These results indicate that the travelling durations of individual cells were estimated within 10 ms and thus the maximal throughput of this developed platform was estimated to be 100 cells per second (refer to Fig. 4). Since the response times of the PMT were in the range of 10 nm, there is a huge room for further improvement in the throughput of the developed system under the condition that the noises of the PMT can be controlled properly.

For the stable domains of the pulses, the obtained values of *I*_f_ can indicate to an extent the numbers of β-actins, which were quantified as 85 ± 24 mv (A549 cells, n_cell_ = 14 228), 76 ± 26 mv (Hep G2 cells, n_cell_ = 35 932), 146 ± 58 mv (MCF 10 A cells, n_cell_ = 16 650) and 130 ± 36 mv (HeLa cells, n_cell_ = 26246), respectively (refer to Fig. 4). The stable domains suggest that the stage of full occupation of deformed cells within the detection region formed by the constriction microchannel and the chrome window. Since the β-actins were extensively expressed intracellularly, it was assumed that there is an even distribution of fluorescence labelled β-actin antibodies within the detection region, which were confirmed by the low standard deviation to average ratios of individual pulses (e.g., 106 ± 8 mv for an A549 cell and 180 ± 16 mv for a MCF 10 A cell).

Thus calibration curves can be obtained by directly flushing solutions of fluorescence labelled β-actin antibodies with a group of concentrations into the detection region. As shown in Supp. Figure 2, in the calibration curve, there is a linear relationship between the intensities of fluorescence (*I*_*f*_) and concentrations of proteins (*C*_*p*_) where the detection range was 0.001–10 μM. Note that since fluorescent data were measured by PMT, fluorescent signals were averaged in the detection domain. Thus, cytosolic proteins without perfect even distributions can also be characterized by the developed platform under the condition that the standard deviation to average ratios of individual pulses are kept low^38^.

Furthermore, although fluorescent intensities are deeply affected by environmental media including pH and ionic strengths, the calibrating curve developed in this study may still function well because of the following reasons. After membrane permeability and staining of antibodies, the solutions of antibody staining dominate the components of cytosolic media. In the step of calibration, the antibodies with fluorescent probes are thoroughly mixed in solutions of cell staining and thus the environmental media in experiments and calibrations were comparable.

Meanwhile, the ratios of 2*T*_*s*_/(*T*_*r*_ + *T*_*d*_) for these four types of cells were quantified as 2.53 ± 1.25 (A549 cells, n_cell_ = 14 242), 1.89 ± 1.32 (Hep G2 cells, n_cell_ = 35 932), 2.32 ± 1.31 (MCF 10 A cells, n_cell_ = 16 650) and 1.66 ± 0.98 (HeLa cells, n_cell_ = 26 246), respectively. Based on the analysis in the section of “Working Principle”, this parameter can be correlated positively with the elongations of individual cells in the constriction microchannel, which can be further translated to cellular diameters (refer to Fig. 4). Note that variations in aspiration pressures, channel geometries, cellular diameters and stiffness, as well as frictions between deformed cells and the walls of constriction microchannels may affect the travelling speeds of cells in the constriction microchannel. Thus, the dimensionless parameter of 2*T*_*s*_/(*T*_*r*_ + *T*_*d*_) rather than cellular travelling durations (e.g., *T*_*s*_, *T*_*r*_, *T*_*d*_) were used for the quantification of cell sizes.

### Counting of Single-Cell β-Actins

**Figure 5:**
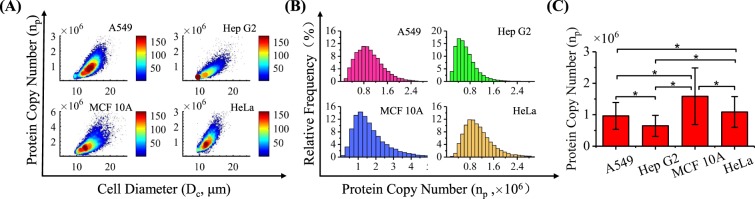
(**A**) Scatter plots of *D*_*c*_ (cell diameter) vs. *n*_*p*_ (numbers of β-actins at the single-cell level) for A549 (n_cell_ = 14 242), Hep G2 (n_cell_ = 35 932), MCF 10 A (n_cell_ = 16 650) and HeLa (n_cell_ = 26 246) cells. (**B**) Distributions and (**C**) histograms of numbers of β-actins at the single-cell level for these four types of cells (*represents differences with statistical significance).

Based on the translation of preliminary fluorescent data and the calibrating curve, diameters of cells (*D*_*c*_) and numbers of β-actins at single-cell level (*n*_*p*_) were obtained as 14.2 ± 1.7 μm and 9.62 ± 4.29 × 10^5^ (A549, n_cell_ = 14 242), 13.0 ± 2.0 μm and 6.46 ± 3.34 × 10^5^ (Hep G2, n_cell_ = 35 932), 13.8 ± 1.9 μm and 1.58 ± 0.90 × 10^6^ (MCF 10 A, n_cell_ = 16 650), and 12.7 ± 1.5 μm and 1.09 ± 0.49 × 10^6^ (HeLa, n_cell_ = 26 246), respectively (refer to Fig. 5).

In order to validate these bright-field microscopic images of suspended cells before they were flushed into the constriction microchannels were recorded and processed to quantify cell diameters as 15.7 ± 2.6 μm (A549, n_cell_ = 394), 13.9 ± 2.5 μm (Hep G2, n_cell_ = 196), 19.9 ± 3.8 μm (MCF 10 A, n_cell_ = 228), and 14.1 ± 2.7 μm (HeLa, n_cell_ = 269). For A549, Hep G2 and HeLa cells, comparable results were obtained based on these two approaches. For MCF 10 A cells, based on the observations of microscopes, the averaged diameters approached the upper detection limitation of this microfluidic device where large cells are difficult to be aspirated through the constriction microchannel. In comparison to large cells, MCF 10 A cells with small diameters are more prone to be aspirated by the constriction microchannel with higher travelling speeds. Thus, it was speculated that the diameters quantified by the fluorescent pulses were derived from MCF 10 A cells with smaller diameters. These studies further suggest the developed microfluidic platform cannot be used to character cells with diameters much larger than the critical width of the constriction microchannel.

In addition, the numbers of single-cell β-actins of A549 cells were estimated as 9.62 ± 4.29 × 10^5^, consistent with 1) 3.57 ± 0.22 × 10^6^ per cells obtained from conventional enzyme linked immunosorbent assays and 2) ~0.96 × 10^6^ at the single-cell level based on previously developed microfluidic platforms^38^. Compared to the results of single-cell analysis, conventional enzyme linked immunosorbent assays located a minute higher number of β-actins per cell, indicating that some portions of β-actins are not exposed in the step of intracellular staining, although intracellular staining has been intensively used for deep phenotyping^11,12^ and signaling state characterization^13–16^. In addition, the single-cell β-actin umbers estimated by the microfluidic platform were ten to one hundred folds of the functional proteins which were previously measured^20^. These results agree with the conventional assumptions that the numbers of β-actins are much higher those of functional proteins.

When the numbers of β-actins at the single-cell level were compared among four types of cells ((9.62 ± 4.29 × 10^5^ (A549), 6.46 ± 3.34 × 10^5^ (Hep G2), 1.58 ± 0.90 × 10^6^ (MCF 10 A), and 1.09 ± 0.49 × 10^6^ (HeLa)), significant differences were located, which suggests that expression differences exist among different cell types of β-actins, and the use of β-actins as the internal controls of western blotting need to be further considered. In addition, for each type of the cell, at the single-cell level, there is also a distribution difference in the expression of β-actins where ratios of the standard deviations to averages were estimated as ~50%, further questioning the proper use of β-actins as the internal control for single-cell analysis.

## Conclusion

This manuscript showed the design, fabrication and characterization of a modified flow cytometry leveraging constriction microchannels, in which both cell sizes and counting of β-actins from 10 000 cells were obtained. Future technical improvements may extend multiplex capabilities, enabling the quantitative analysis of multiple types of proteins. In addition, this platform will be used to characterize key proteins such as p53 at the single-cell level, facilitating the study of tumour heterogeneity.

## Electronic supplementary material

Supplementary Materials

## Data Availability

All data generated or analysed during this study are included in this published article.
