# Supplementary material for: A Microfluidic Fluorescent Flow Cytometry Capable of Quantifying Cell Sizes and Numbers of Specific Cytosolic Proteins
Source: Sci Rep. 2018 Sep 21;8:14229. doi: 10.1038/s41598-018-32333-1 (PMC6155059; doi:10.1038/s41598-018-32333-1)
Supplement: Supplementary file 1 — Supplementary Materials [file 41598_2018_32333_MOESM1_ESM.docx]

**Supplementary Materials**

**A Microfluidic Fluorescent Flow Cytometry Capable of Quantifying Cell Sizes and Numbers of Specific Cytosolic Proteins**

Xiufeng Li^1,2^*, Beiyuan Fan^1,2^*, Lixing Liu^1,2^, Deyong Chen^1,2^, Shanshan Cao^3^, Dong Men^3^**, Junbo Wang^1,2^**, and Jian Chen^1,2^**

­^1^State Key Laboratory of Transducer Technology, Institute of Electronics, Chinese Academy of Sciences, Beijing, P.R. China

^2^University of Chinese Academy of Sciences, Beijing, P.R. China

­^3^State Key Laboratory of Virology, Wuhan Institute of Virology, Chinese Academy of Sciences, Wuhan, Hubei Province, P.R. China

*Co-First Authors

**Co-Corresponding Authors:

Dong Men (d.men@wh.iov.cn), Junbo Wang ([jbwang@mail.ie.ac.cn](mailto:jbwang@mail.ie.ac.cn)) and Jian Chen (chenjian@mail.ie.ac.cn)

Supplementary Figure 1: Schematic of fabricating microdevices for the simultaneous quantification of cell sizes and specific intracellular proteins. The PDMS device was fabricated leveraging conventional soft lithography including key steps of (a) SU-8 5 spin coating and exposure without development, (b) SU-8 25 spin coating, exposure with alignment, (c) development, (d) PDMS molding and (e) peeled PDMS with through holes punched. The fabrication of the chrome window was based on conventional hard lithography, including key steps of (f) chrome deposition, (g) photoresist spin coating and exposure, (h) development, (i) chrome etching and (j) the coating of a thin layer of fully cured PDMS. In the end, after plasma treatment, the PDMS layer and the chromium layer were bonded together ((k) and (l)).

Supplementary Figure 2: The obtained calibration curve where a linear correlation between the concentrations of fluorescence labelled antibodies and the fluorescent intensities was located.
